# Exposed Dentin: Influence of Cleaning Procedures and Simulated Pulpal Pressure on Bond Strength of a Universal Adhesive System

**DOI:** 10.1371/journal.pone.0169680

**Published:** 2017-01-12

**Authors:** Simon Flury, Anne Peutzfeldt, Patrick R. Schmidlin, Adrian Lussi

**Affiliations:** 1 Department of Preventive, Restorative, and Pediatric Dentistry, School of Dental Medicine, University of Bern, Bern, Switzerland; 2 Division of Periodontology and Peri-Implant Diseases, Clinic of Preventive Dentistry, Periodontology, and Cariology, Center of Dental Medicine, University of Zurich, Zurich, Switzerland; Nanjing Medical University, CHINA

## Abstract

**Purpose:**

To compare various pre-treatments serving as cleaning procedures of dentin on the bond strength of resin composite promoted by a universal adhesive system applied either in the absence or presence of simulated pulpal pressure.

**Materials and Methods:**

Prior to application of the adhesive system (Scotchbond Universal) and resin composite (Filtek Z250), ground dentin surfaces were given one of five pre-treatments either without or with simulated pulpal pressure: 1) no pre-treatment, adhesive system in “self-etch” mode, 2) phosphoric acid etching, adhesive system in “total-etch” mode, 3) polishing with pumice on prophylaxis cup, 4) air abrasion with AIR-FLOW PLUS powder, 5) air abrasion with AIR-FLOW PERIO powder; n = 20/group of pre-treatment. After storage (37°C, 100% humidity, 24 h), micro shear bond strength was measured and data analyzed with parametric ANOVA including Bonferroni-Holm correction for multiple testing followed by Student’s t tests (significance level: α = 0.05).

**Results:**

The ANOVA found type of pre-treatment and simulated pulpal pressure to have no significant effect on dentin bond strength. The explorative post-hoc tests showed a negative effect of simulated pulpal pressure for phosphoric acid etching (adhesive system in “total-etch” mode; p = 0.020), but not for the other four pre-treatments (all p = 1.000).

**Conclusion:**

Air abrasion with powders containing either erythritol and chlorhexidine (AIR-FLOW PLUS) or glycine (AIR-FLOW PERIO) yielded dentin bond strengths similar to no pre-treatment, phosphoric acid etching, or polishing with pumice. Simulated pulpal pressure reduced the bond strength only when the self-etch adhesive system was used in total-etch mode.

## Introduction

Exposed dentin is a frequently occurring clinical problem caused by abrasive and/or erosive forces [1]. For some patients, the exposed dentin has no clinical implications whereas for others, it may cause dentin hypersensitivity [1]. In the initial stage, non-invasive individual prophylactic home-care approaches such as fluoride rinsing solutions, fluoride toothpastes, or fluoride varnishes should be the first choice of treatment [1,2]. In an advanced stage, sealing of the open dentin tubules can be established with a mechanical barrier based on adhesive bonding such as the application of adhesive systems as dentin desensitizers. Finally, resin composite restorations may be indicated in an even more advanced stage characterized by loss of dentin substance [1,3].

However, durable adhesive bonding to exposed dentin may be hampered since this dentin is prone to alteration by mechanical and/or chemical modifications [4], often combined with the presence of bacteria in the biofilm invariably found on non-shedding surfaces [5]. Consequently, to permanently seal the dentin, the surface should be properly cleaned in order to allow for adequate adhesive bonding [6]. The most common cleaning procedure is the use of prophylaxis brushes or cups and an abrasive paste such as pumice. In recent years, different methods and devices have been developed and recommended to help clinicians e.g. remove bacteria on the dentin surface and/or in periodontal pockets. Amongst others, newer powders containing erythritol and chlorhexidine (such as AIR-FLOW PLUS; Electro Medical Systems SA [EMS]) or glycine (such as AIR-FLOW PERIO; EMS) have been marketed. These two newer powders can be applied using portable air abrasion handpieces and have been shown to be less invasive than hand scaling, ultrasonic scaling, or air abrasion with traditional sodium bicarbonate powder [7,8]. Bearing in mind that sodium bicarbonate powder impairs the dentin bonding performance of adhesive systems [9–11] and that exposed dentin may be of varying nature (e.g. with open dentin tubules in the case of dentin hypersensitivity, with alterations due to mechanical and/or chemical modifications, and in some cases with a biofilm), it might be indicated to use one of the two newer powders as a cleaning procedure before application of adhesive systems as dentin desensitizers or before placement of resin composite restorations. However, there is no information about how pre-treatment of dentin with these two powders affects bond strength of adhesive systems and resin composite. Consequently, the aim of the present study was to investigate bond strength of resin composite and a universal adhesive system (Scotchbond Universal; 3M ESPE) to dentin under different conditions, including absence or presence of simulated pulpal pressure since clinically, pulpal pressure may lead to moisture in the form of dentinal fluid, which has previously been shown to potentially compromise bonding of adhesive systems [12,13].

The null hypotheses were that bond strength would be the same for all groups 1) irrespective of pre-treatment of the dentin and 2) irrespective of simulated pulpal pressure.

## Materials and Methods

### Preparation of dentin specimens

A total of 200 dentin specimens (n = 20/group; 10 groups [5 pre-treatments without/with simulated pulpal pressure]) were prepared from extracted human molars. Before extraction, patients had been informed about the use of the teeth for research purposes, and verbal consent had been obtained. After extraction, the teeth were pooled and the local ethics committee categorizes the pooled teeth as “irreversibly anonymized bio-bank” and thus, no previous ethical approval was necessary. The molars were cleaned with a scaler and stored in 2% chloramine solution in the refrigerator (4°C) until needed. For preparation of dentin specimens, the molars were apically shortened with a water-cooled diamond saw (IsoMet Low Speed Saw, Buehler; Lake Bluff, IL, USA) and ground from the buccal surface until in dentin, resulting in flat dentin surfaces without caries, restorations, or exposure of the pulp. Grinding was performed under water-cooling with grit #220 followed by grit #500 silicon carbide (SiC) papers (Struers; Ballerup, Denmark) on a Struers LaboPol-21 grinding machine (Struers). Subsequently, the molars were embedded in self-curing acrylic resin (Paladur, Heraeus Kulzer; Hanau, Germany) in cylindrical stainless steel molds. After removal of the molds, one half of the dentin specimens (n = 100), intended for use without simulated pulpal pressure, was subsequently kept in storage solution (pH 7) according to Zero [14] and in the refrigerator (4°C) until needed. The other half of the dentin specimens (n = 100), intended for use with simulated pulpal pressure, was apically trepanned through the self-curing acrylic resin with a diamond bur, and the tissue of the pulpal chamber was retrogradically removed. Stainless steel tubes (outer diameter 1.5 mm; aperture 1 mm) were inserted through the trepanation and fixed with resin (LC Block-Out Resin, Ultradent Products; South Jordan, UT, USA) in analogy to previous studies [15,16]. These dentin specimens were subsequently stored until needed as explained for the dentin specimens intended for use without simulated pulpal pressure.

### Preparation of micro shear bond strength (μSBS) specimens

One hour before preparation of μSBS specimens, the dentin specimens were retrieved from the refrigerator and kept in tap water at room temperature. The pulpal chamber of dentin specimens used with simulated pulpal pressure was rinsed through the stainless steel tubes with 17% EDTA-solution (pH 8) for 15 s followed by rinsing with tap water for 15 s. Then, the dentin surface of all specimens was re-ground under water-cooling for 5 s on grit #500 SiC abrasive papers (Struers) to obtain a standardized smear layer. The grit #500 SiC abrasive paper was changed after grinding of 10 specimens. Dentin specimens used with simulated pulpal pressure were connected by their stainless steel tubes to a custom made hydrostatic pressure device filled with deionized water as previously described [15,16]. Subsequently, the dentin specimens underwent one of five pre-treatments either without or with simulated pulpal pressure as listed in Table 1, leading to a total of ten groups (n = 20/group): Group SE (no pre-treatment of dentin; adhesive system used in “self-etch” mode without simulated pulpal pressure), Group SE-P (no pre-treatment of dentin; adhesive system used in “self-etch” mode with simulated pulpal pressure), Group TE (pre-treatment of dentin with phosphoric acid; adhesive system used in “total-etch” mode without simulated pulpal pressure), Group TE-P (pre-treatment of dentin with phosphoric acid; adhesive system used in “total-etch” mode with simulated pulpal pressure), Group PUM (pre-treatment of dentin with pumice [Nupro Prophylaxis Paste “Stain Removal” and prophylaxis cups Crescent Prophy Cups RA Webbed, DENTSPLY Professional/Rinn; York, PA, USA] without simulated pulpal pressure), Group PUM-P (pre-treatment of dentin with pumice [Nupro Prophylaxis Paste “Stain Removal” and prophylaxis cups Crescent Prophy Cups RA Webbed, DENTSPLY Professional/Rinn] with simulated pulpal pressure), Group PLUS (pre-treatment of dentin with AIR-FLOW PLUS powder [Lot No: 140131] and the AIR-FLOW HANDY 3.0 PERIO handpiece [EMS; Nyon, Switzerland] without simulated pulpal pressure), Group PLUS-P (pre-treatment of dentin with AIR-FLOW PLUS powder [Lot No: 140131] and the AIR-FLOW HANDY 3.0 PERIO handpiece [EMS] with simulated pulpal pressure), Group PERIO (pre-treatment of dentin with AIR-FLOW PERIO powder [Lot No: 1301171] and the AIR-FLOW HANDY 3.0 PERIO handpiece [EMS] without simulated pulpal pressure), Group PERIO-P (pre-treatment of dentin with AIR-FLOW PLUS powder [Lot No: 1301171] and the AIR-FLOW HANDY 3.0 PERIO handpiece [EMS] with simulated pulpal pressure).

**Table 1:** The five pre-treatments used prior to application of the adhesive system.

Groups	Simulated pulpal pressure	Treatment steps	Time (s)
Group SE	No	(No pre-treatment of dentin)	
Group SE-P	Yes	1) Definition of bonding area on dentin with perforated self-adhesive tape^1^	
Group TE	No	1) Definition of bonding area on dentin with perforated self-adhesive tape^1^	
Group TE-P	Yes	2) Pre-treatment of dentin with phosphoric acid (Scotchbond Universal Etchant, Lot No: 569779)	15
		3) Water-spray	10
		4) Gentle air-dry	
Group PUM	No	1) Pre-treatment of dentin with pumice, prophylaxis cups with 1500 rotations per minute	10
Group PUM-P	Yes	2) Water-spray	10
		3) Gentle air-dry	
		4) Definition of bonding area on dentin with perforated self-adhesive tape^1^	
Group PLUS	No	1) Pre-treatment of dentin with AIR-FLOW PLUS powder, working distance ~10 mm;	10
Group PLUS-P	Yes	no rinsing with water	
		2) Definition of bonding area on dentin with perforated self-adhesive tape^1^	
Group PERIO	No	1) Pre-treatment of dentin with AIR-FLOW PERIO powder, working distance ~10 mm;	10
Group PERIO-P	Yes	no rinsing with water	
		2) Definition of bonding area on dentin with perforated self-adhesive tape^1^	

^1^: (diameter of the perforation ~2 mm)

After pre-treatment and definition of the bonding area (Table 1), the adhesive system Scotchbond Universal (3M ESPE; Neuss, Germany; Lot No: 591396) was applied on the dentin surface within the defined bonding area, rubbed in with an applicator tip for 20 s, gently air-dried for 5 s, and light-cured for 10 s. Subsequently, a split Teflon mold (inner diameter 1.5 mm ≈ bonding area 1.8 mm^2^; height 2 mm) was clamped to the dentin surface and filled with resin composite (Filtek Z250, 3M ESPE; St. Paul, MN, USA; shade A3, Lot No: N657007) and the resin composite was light-cured for 20 s. All light-curing was performed with an LED curing unit (Demi, Kerr Corporation; Middleton, WI, USA) with a mean light power density of 1600 mW/cm^2^ (measured with the MARC PS, BlueLight Analytics Inc.; Halifax, NS, Canada). Dentin specimens used with simulated pulpal pressure were then disconnected from the custom made hydrostatic pressure device.

The resulting μSBS specimens were placed in black photo-resistant boxes in order to avoid any additional effect of ambient light on the initial polymerization process. Five minutes after completion of light-curing and at room temperature, the specimens were freed from the Teflon mold. The specimens were stored in the black photo-resistant boxes in an incubator (Memmert UM 500, Memmert & Co.; Schwabach, Germany) at 37°C and 100% humidity for 24 h.

### μSBS Testing and failure mode determination

After storage, specimens were subjected to μSBS testing as previously described [17] by use of a wire (stainless steel, diameter 0.6 mm) in a universal testing machine (Zwick Z1.0 TN, Zwick; Ulm, Germany) at a cross-head speed of 1 mm/min. The maximum force (F_max_ [N]) was recorded (testXpert software V9.0, Zwick) and the μSBS values (MPa) were calculated (F_max_ [N] / bonding area [mm^2^]) resulting in 20 μSBS values per group for statistical analysis.

After μSBS testing, the failure mode of each specimen was determined under a stereomicroscope (Leica ZOOM 2000, Leica; Buffalo, NY, USA) at 40× magnification and classified as 1) cohesive failure in dentin, 2) adhesive failure at dentin—adhesive interface, 3) adhesive failure at adhesive—resin composite interface, 4) cohesive failure in resin composite, or 5) mixed failure (combinations of failure modes 1) to 4)).

### Statistical analyses

A normal QQ-plot and the Shapiro Wilk’s test (p = 0.336) showed that the μSBS values were normally distributed and thus, μSBS values were analyzed with a parametric ANOVA and the p-values were corrected with Bonferroni-Holm adjustment for multiple testing. For a further explorative analysis of an effect of simulated pulpal pressure within a given pre-treatment, Student’s t tests for two unpaired samples were performed as post-hoc tests. All calculations were performed with R version 3.2.2 (The R Foundation for Statistical Computing; Vienna, Austria; www.R-project.org), the level of significance having been set at α = 0.05. Failure modes after μSBS testing were analyzed descriptively.

## Results

**Fig 1:**
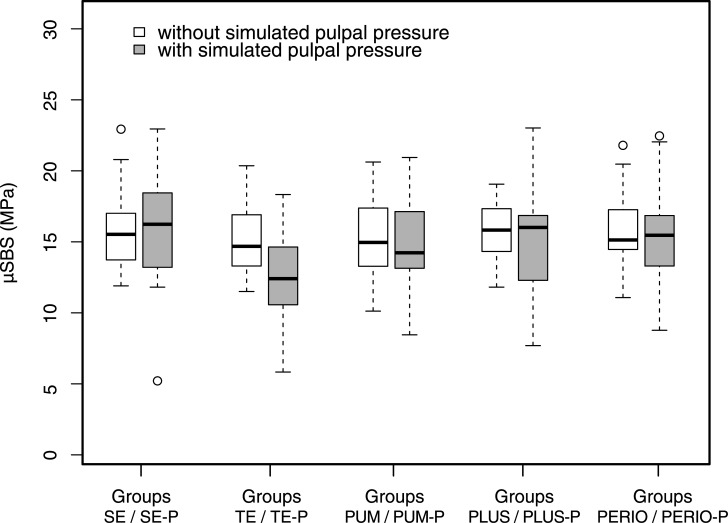
Micro shear bond strength (μSBS [MPa]) of the ten groups (five pre-treatments without/with simulated pulpal pressure).

Micro shear bond strength (μSBS [MPa]) of the ten groups (i.e. of the five pre-treatments without/with simulated pulpal pressure) is shown in Fig 1 and the mean values (standard deviations) ranged from 12.5 MPa (3.19) to 16.1 MPa (4.22).

The ANOVA showed no significant effect of the factor “pre-treatment” (p = 1.000), no significant effect of the factor “simulated pulpal pressure” (p = 0.162), and no significant interaction of the two factors (p = 1.000). However, the post-hoc tests for an explorative analysis of an effect of simulated pulpal pressure within a given pre-treatment found the μSBS of Group TE-P to be significantly lower than that of Group TE (p = 0.020). Within the other four pre-treatments (i.e. between Group SE and SE-P, PUM and PUM-P, PLUS and PLUS-P, or PERIO and PERIO-P), μSBS did not significantly differ (all p = 1.000).

The distribution of failure modes after μSBS testing is shown in Table 2. The predominant failure mode for all ten groups was adhesive failure at the dentin—adhesive interface. Group SE (without simulated pulpal pressure) and Group SE-P (with simulated pulpal pressure) showed a similar percentage of cohesive failures in dentin. Compared to Groups SE and SE-P, all other groups generally showed a markedly lower percentage of cohesive failures in dentin. All groups with simulated pulpal pressure except Groups SE-P and PLUS-P showed no cohesive failure in dentin. All ten groups showed a similar percentage of mixed failures, the vast majority of the mixed failures consisting of a combination of cohesive failures in dentin and adhesive failure at the dentin—adhesive interface.

**Table 2:** Distribution of failure modes after micro shear bond strength testing (n = 20/group).

Groups	1) Cohesive failure in dentin (%)	2) Adhesive failure at dentin—adhesive interface (%)	3) Adhesive failure at adhesive—resin composite interface (%)	4) Cohesive failure in resin composite (%)	5) Mixed failure (%)
Group SE	20	60	0	0	20
Group SE-P	25	55	0	0	20
Group TE	5	80	0	0	15
Group TE-P	0	85	0	0	15
Group PUM	10	70	0	0	20
Group PUM-P	0	80	0	0	20
Group PLUS	15	60	0	0	25
Group PLUS-P	5	75	0	0	20
Group PERIO	25	55	0	0	20
Group PERIO-P	0	85	0	0	15

## Discussion

The current study compared various pre-treatments of dentin on the bond strength of resin composite promoted by a universal adhesive system, which was applied either in the absence or presence of simulated pulpal pressure. No differences in bond strength were found between the pre-treatments and thus, the first hypothesis cannot be rejected.

**Fig 2:**
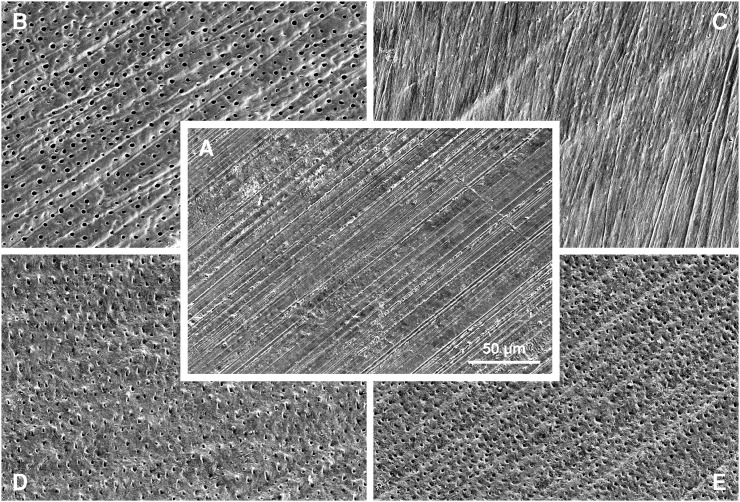
Scanning electron micrographs of representative dentin surfaces after no pre-treatment (A), pre-treatment with phosphoric acid (B), pre-treatment with pumice (C), pre-treatment with AIR-FLOW PLUS powder (D), and pre-treatment with AIR-FLOW PERIO powder (E). Gold-palladium sputter coating (100 s, 50 mA; Balzers SCD 050, Balzers, Liechtenstein), 500× magnification (JEOL JSM6010PLUS/LV, JEOL, Tokyo, Japan).

Since air-powder polishing is commonly used in clinical practice, it is encouraging that the two AIR-FLOW powders yielded bond strengths of the same magnitude as did traditional polishing with pumice on a prophylaxis cup, especially considering that previous studies have found air-powder polishing with sodium bicarbonate to impair dentin bonding performance of self-etch adhesive systems [9–11]. As with AIR-FLOW PERIO in the present study, pre-treatment with another glycine-containing powder (Clinpro Glycine Prophy Powder) has previously been shown not to cause any reduction in dentin bond strength when tested with a broad range of adhesive systems of all categories [9]. The fact that the two AIR-FLOW powders resulted in identical bond strengths and failure mode distributions may be a reflection of their similar dentin surface morphology with removal of the smear layer and smear plugs and partial opening of the dentin tubules to a similar degree (Fig 2).

As the name implies, Scotchbond Universal is marketed as a universal adhesive, i.e. an adhesive system that can be used in “self-etch” as well as in “total-etch” mode, e.g. depending on the clinician’s assessment of each specific clinical case. The present study found no difference in bond strength between the two application modes. This finding not only corroborates the claims of the manufacturer, improving clinical versatility, but it is also in harmony with the results of previous studies [18–20]. The phosphoric acid applied according to the “total-etch” mode results in removal of the smear layer and smear plugs and in opening of the dentin tubules (Fig 2). Besides, complete demineralization of the dentin to a depth of 3–5 μm has previously been observed [21]. In contrast, when used according to the “self-etch” mode the demineralization depends on the acidity of the adhesive system per se [21]. With a pH of around 2, Scotchbond Universal classifies as a mild self-etch adhesive system [21] and is known to result in only partial demineralization of the dentin and in the formation of a hybrid layer less than 1 μm thick [21,22]. The fact that the two different application modes resulted in similar bond strengths is in accordance with a previous study on Scotchbond Universal [19] and implies that effective, short-term bonding to dentin can be obtained by at least two approaches. Compared to the “total-etch” mode, the “self-etch” mode seems to present several clinical advantages: a simplified application protocol, a less invasive pre-treatment, and the absence of a water-rinsing step following phosphoric acid etching and, more importantly, the consequent lack of need to dry the etched dentin—a step which is known to be critical for the bonding performance [23].

Not only was there no positive effect on bond strength of including a phosphoric acid etching step in the application of Scotchbond Universal (i.e. the “total-etch” mode) as compared to the “self-etch” mode, but phosphoric acid etching actually caused a reduction in bond strength when specimens had been prepared in the presence of simulated pulpal pressure, leading to partial rejection of the second hypothesis. This finding is in accordance with that of Silva et al. [12] and may be explained as follows: Simulated pulpal pressure implies a constant intrinsic wetness of the dentin. If the presence of water did not hamper infiltration of the self-etch adhesive system to its reported depth of less than 1 μm [21,22], it may have hindered complete infiltration of the adhesive system into the 5 μm thick zone of totally demineralized dentin caused by phosphoric acid etching and thus compromised the quality of the hybrid layer and of the adhesive bond. Incomplete evaporation of the ethanol solvent in the presence of excessive moisture may have acted as an aggravating factor [24]. Although it must be kept in mind that the ANOVA showed no significant effect of the factor “simulated pulpal pressure” and that the significant difference in bond strength between Group TE and TE-P was the result of explorative post-hoc tests, this finding indicates that Scotchbond Universal should be used in “self-etch” mode on dentin. Consequently, if “selective” phosphoric acid etching of enamel is indicated, the clinician should strive to limit the etching to the enamel part of the cavity.

With regard to the methods applied, the present in vitro study used a micro shear bond strength test to assess the bond strength to dentin. Shear bond strength tests have been criticized for having limited discrimination power compared to e.g. microtensile bond strength tests. However, shear bond strength tests are well-established and do not require laborious, traumatic processing that may result in pre-test failures. In the present study, no pre-test failures occurred and no significant differences between the groups were found. The latter finding might reflect a limited discrimination power of the test method. On the other hand, since the results are in corroboration with those of other studies, some of which used microtensile bond strength tests, the results most likely reflect that there are no “true” differences between the pre-treatments. Irrespective of pre-treatment, the predominant failure mode by far was adhesive failure at the dentin-adhesive interface. However, the fact that Scotchbond Universal when used in “self-etch” mode without any pre-treatment caused the most cohesive failures in dentin, i.e. also more than use in “total-etch” mode, may reflect a certain superiority of this adhesive interface, e.g. resulting increased stability of the self-etch bond compared to the total-etch bond as have been reported in previous studies [25,26]. Evidently, an adhesive treatment must not only be able to provide effective, immediate bonding, but this effective bonding must also be durable. Thus, the resistance to long-term water storage of the bonds resulting from air abrasion with the AIR-FLOW powders should be investigated in future studies.

## Conclusions

There were no differences in dentin bond strength among the pre-treatment cleaning procedures investigated. Thus, air abrasion with powders containing either erythritol and chlorhexidine or glycine yielded similar dentin bond strengths as did no pre-treatment or polishing with pumice. The presence of simulated pulpal pressure during specimen preparation reduced the bond strength only when the self-etch adhesive system was used in total-etch mode.

## Supporting Information

S1 File Raw data. Spreadsheet (Excel file) of micro shear bond strength (μSBS) values of the ten groups (i.e. of the five pre-treatments without/with simulated pulpal pressure).
(XLS)
